# Phospholipase C Zeta 1 (PLCZ1): The Function and Potential for Fertility Assessment and In Vitro Embryo Production in Cattle and Horses

**DOI:** 10.3390/vetsci10120698

**Published:** 2023-12-11

**Authors:** Raul A. Gonzalez-Castro, Elaine M. Carnevale

**Affiliations:** Equine Reproduction Laboratory, Department of Biomedical Sciences, Colorado State University, Fort Collins, CO 80523, USA; raul.gonzalez_castro@colostate.edu

**Keywords:** bull, fertility, ICSI, oocyte, PLCZ1, sperm, stallion

## Abstract

**Simple Summary:**

After sperm–oocyte fusion, oocytes are activated by a sperm factor for fertilization and embryo development success. Evidence proposes that sperm-borne phospholipase C Zeta 1 (PLCZ1) as an activating factor. The alteration of the gene or protein expression of PLCZ1 results in low or no fertility. In human assisted reproduction, PLCZ1 characterization is of essential importance when oocyte activation failures are linked to male infertility. However, little is known about the importance of PLCZ1 for in vivo and in vitro fertility outcomes in cattle and horses, particularly as the commercial use of assisted fertilization grows. In horses, intracytoplasmic sperm injection (ICSI) is the elective method for assisted fertilization. Sperm PLCZ1 amount exhibits a large variation from stallion to stallion which can influence fertility outcomes. Stallion sperm samples showing low PLCZ1 consistently result in lower fertility after ICSI. In contrast, ICSI is not efficient in cattle due to the inconsistent ability of bull sperm to activate bovine oocytes. Interestingly, the experimental microinjection of exogenous PLCZ1 is able to rescue oocytes that failed to activate, even reaching live birth. PLCZ1 has the potential to be used as a diagnostic and predictive tool for sperm-fertilizing capability and therapeutic treatment for oocyte failure under assisted fertilization.

**Abstract:**

Phospholipase C Zeta 1 (PLCZ1) is considered a major sperm-borne oocyte activation factor. After gamete fusion, PLCZ1 triggers calcium oscillations in the oocyte, resulting in oocyte activation. In assisted fertilization, oocyte activation failure is a major cause of low fertility. Most cases of oocyte activation failures in humans related to male infertility are associated with gene mutations and/or altered PLCZ1. Consequently, PLCZ1 evaluation could be an effective diagnostic marker and predictor of sperm fertilizing potential for in vivo and in vitro embryo production. The characterization of PLCZ1 has been principally investigated in men and mice, with less known about the PLCZ1 impact on assisted reproduction in other species, such as cattle and horses. In horses, sperm PLCZ1 varies among stallions, and sperm populations with high PLCZ1 are associated with cleavage after intracytoplasmic sperm injection (ICSI). In contrast, bull sperm is less able to initiate calcium oscillations and undergo nuclear remodeling, resulting in poor cleavage after ICSI. Advantageously, injections of PLCZ1 are able to rescue oocyte failure in mouse oocytes after ICSI, promoting full development and birth. However, further research is needed to optimize PLCZ1 diagnostic tests for consistent association with fertility and to determine whether PLCZ1 as an oocyte-activating treatment is a physiological, efficient, and safe method for improving assisted fertilization in cattle and horses.

## 1. Introduction

Oocyte activation is initiated when a fertilizing sperm delivers sperm-borne oocyte-activating factors into the oocyte cytoplasm [1,2,3]. Experimental evidence indicates that phospholipase C zeta 1 (PLCZ1) meets the criteria of the soluble oocyte-activating factor responsible for initiating calcium (Ca^2+^) oscillations after gamete fusion at mammalian fertilization [4,5,6,7,8,9,10,11,12,13]. The fertilizing sperm releases PLCZ1 into the ooplasm, triggering Ca^2+^ oscillations via the inositol trisphosphate (InsP3) signaling pathway through the hydrolysis of organelle membrane-bound substrates [5,14]. The oscillatory pattern of intracellular Ca^2+^ rises activates pathways that result in the resumption and completion of meiosis II, the extrusion of the second polar body, and the initiation of preimplantation development [15]. In fertilized and successfully activated oocytes, Ca^2+^ oscillations also regulate short- and long-term embryonic developmental events [2,16], confirming the essential role of PLCZ1 for fertilization and embryo competence.

Mice have been used as the primary research model for the characterization of PLCZ1 and understanding its role in oocyte activation [4,5,9,12,17,18,19,20,21,22,23]. Most clinical investigations of PLCZ1 have been performed in humans due to the importance and implications for oocyte activation failure after in vitro fertilization (IVF) and intracytoplasmic sperm injection (ICSI). The importance of oocyte activation is especially notable after ICSI, as the initial stages of embryo development can be directly observed. The relationship between male infertility and PLCZ1 in men has been confirmed in several clinical and experimental studies. The absence, reduction, and abnormal localization of PLCZ1 in human sperm or mutations of PLCZ1 in men are associated with ICSI failure, low fertilization rates, and impaired embryo development [13,19,24,25,26,27,28,29,30,31,32,33,34,35,36,37,38,39,40,41,42,43,44,45,46]. In this context, the assessment of PLCZ1 in sperm samples used for IVF and ICSI has diagnostic and predictive value for fertility in men, but similar studies are lacking in animals.

The limited research on the role of PLCZ1 in male fertility in livestock species is possibly attributed to the predominance of natural fertilization. During natural cover or artificial insemination, PLCZ1 alterations can be compensated due to the large total sperm amount and sperm selection resulting from the female reproductive tract–sperm interaction that removes defective sperm [47,48]. In *PLCZ1*-knockout mice, natural fertilization can also occur in the absence of sperm PLCZ1 via an alternative PLCZ1-independent mechanism [49,50]. During in vitro fertilization, oocyte activation failure after sperm injection is probably a major cause of fertility failure in horses and humans [51,52,53]. Sperm from some stallions result in consistently good embryo development rates after ICSI, while sperm from other stallions consistently result in poor or no embryo development [54,55,56], suggesting a difference in oocyte activating potentials. In equine ICSI, plasma membrane integrity is found to be the sperm attribute most highly associated with embryo development and pregnancy after ICSI when compared to sperm morphology and DNA integrity [57]. These findings suggest that a sperm membrane component, potentially PLCZ1, is required for oocyte activation and the initiation of early embryo development after equine ICSI.

In addition to men, identification and full or partial characterization of PLCZ1 have been performed in sperm from boars [5,58,59], buffalos [60], bulls [13,61,62,63,64], cats [65], hamsters [66,67], stallions [68,69], monkeys [4], mice [5,62,70] and rats [71]. However, the role of PLCZ1 in fertility outcomes has been poorly documented in livestock species, especially when assisted reproductive technologies are commercially applied for the in vitro production of embryos in cattle and horses through IVF and ICSI, respectively. In this review, we will discuss recent findings in understanding the importance of PLCZ1 in sperm fertilizing ability and initiation of oocyte activation in mammalian oocytes, and we will review the impact of recent biotechnology on fertility outcomes after assisted reproductive technologies in cattle and horses.

## 2. The Structure and Domain Organization of PLCZ1

Phospholipase C zeta 1 (PLCZ1) is a sperm-specific, 1-phosphatidylinositol 4,5-bisphosphate phosphodiesterase that has been identified in the sperm of men, livestock species, and other mammals [4,5,13,58,59,60,62,63,64,65,68,69,70,71]. PLCZ1 is the smallest phospholipase C isoform. The molecular weight of PLCZ1 is similar across mammalian species, at 70 to 75 kDa [4,5,11,65,71].

**Figure 1:**
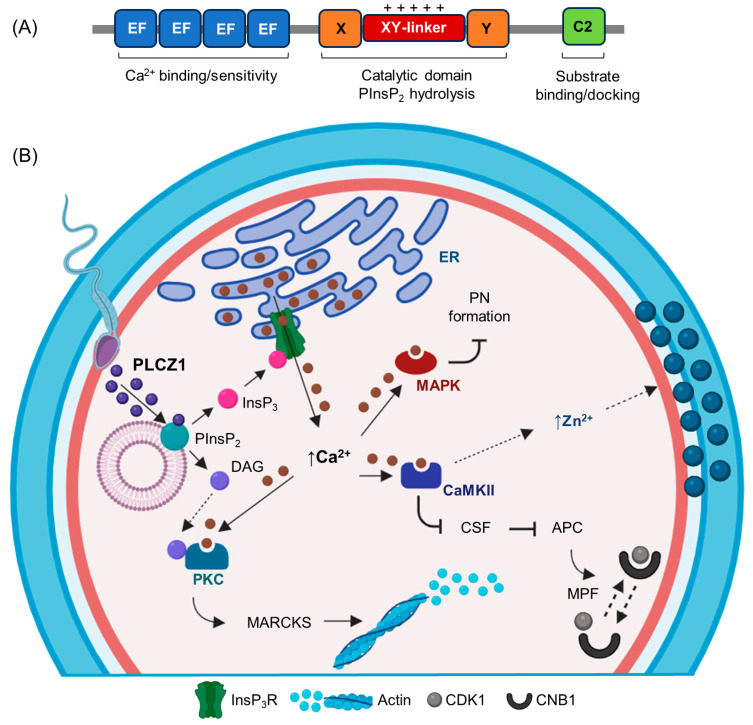
Representation of domain structure and function of PLCZ1. (**A**) PLCZ1 is formed by four tandem EF hand-like domains, X and Y catalytic domains connected by XY-linker region, and a single C2 domain. (**B**) After gamete fusion, PLCZ1 is released into the ooplasm, targeting intracellular membrane-bound phosphatidylinositol 4,5-biphosphate (PInsP2) to be catalyzed into Inositol-1,4,5-triphosphate (InsP3) and diacylglycerol (DAG). InsP3 binds to a specific receptor in the endoplasmic reticulum (ER), causing Ca^2+^ release. Ca^2+^ upregulates calcium/calmodulin-dependent protein kinase II (CaMKII) which inhibits cytostatic factor EMI2 (CSF), liberating the anaphase-promoting complex/cyclosome (APC) to degrade Cyclin B1 (CNB1) from maturation-promoting factor complex (MPF), promoting meiotic resumption. Ca^2+^ also upregulates protein kinase C (PKC), initiating actin breakdown via myristoylated alanine-rich C kinase substrate (MARCKS) for cortical granule exocytosis. Simultaneously, Ca^2+^ inactivates mitogen-activated protein kinase (MAPK), allowing pronucleus (PN) formation. CDK1: Cyclin-dependent kinase I. InsP3R: InsP3 receptor.

The protein organization of PLCZ1 is formed by four tandem EF hand-like domains in the N-terminal, X and Y catalytic domains connected by an XY-linker region, and a single C2 domain in the C-terminal (Figure 1A). PLCZ1 has the most elementary domain organization when compared to other PLCs, lacking N-terminal pleckstrin and Src homology domains [4,5,22,23,72,73,74]. Each domain has a determined role for the PLCZ1 function. The EF hand-like domains confer sensitivity and binding ability for Ca^2+^ and participate in the nuclear translocation of PLCZ1 in mouse oocytes [75]. The X and Y catalytic domains contain a basic cluster of amino acids that anchor to phosphatidylinositol-4,5-bisphosphate [PIns(4,5)P2], which is responsible for enzymatic activity [5,72]. The XY-linker region is positively charged and targets intracellular membranes via direct electrostatic interactions with negatively charged PI(4,5)P2 [22,23,72,73]. The C2 domain distinctly targets phospholipids in the membrane of intracellular vesicles by interacting with phosphoinositides. The C2 domain also plays an important role in propagating Ca^2+^ waves through the ooplasm [9,14,22,26,76,77]. Although the structural domain organization of PLCZ1 has been characterized, the crystal structure of PLCZ1 has yet to be defined [76]. A determination of the three-dimensional PLCZ1 structure will improve understanding of the precise mechanism of PLCZ1 action during oocyte activation.

Structural differences or alterations in PLCZ1 can modify its enzymatic function in different species. In mouse oocytes, the complete or partial deletion of both the EF hand-like and C2 domains has no effect on in vitro PLCZ1 enzymatic activity, but the deletions abolish oocyte activation in vivo [9,78]. In porcine sperm, PLCZ1 cleaved within its XY-linker region remains functional, suggesting that an intact XY-linker region is not essential for the enzymatic activity of porcine sperm [79]. In fact, the majority of the PLCZ1 gene mutations described in men are located in the X and Y catalytic domains, which impair PIns(4,5)P2 hydrolysis and downregulate Ca^2+^ oscillatory activity [30,76]. A fully defined and determined structure is essential for understanding the precise mechanism and function of PLCZ1 during fertilization among species and establishing diagnostic and predictive tools for fertility.

## 3. The Oocyte Activation Function and Sperm Localization of PLCZ1

Gamete fusion is essential for Ca^2+^ release and, consequently, oocyte activation. In mouse oocytes, Ca^2+^ oscillations begin 1–3 min after sperm–oocyte fusion [80]. Under low pH conditions, gamete fusion is prevented, eliminating the initial Ca^2+^ release [81,82]. A simple explanation for these findings is that sperm contains a “soluble” component that diffuses into the ooplasm after gamete fusion, causing Ca^2+^ release. The term “soluble” means that the sperm factor is capable of diffusing throughout the ooplasm to initiate Ca^2+^ release [83]. The term “soluble” does not denote that the sperm factor is kept in a soluble state within the sperm, and it cannot be extracted from sperm into an aqueous solution.

Among PLC isoforms, sperm-specific PLCZ1 displays a unique potency to trigger Ca^2+^ oscillations for oocyte activation; this is attributed to its unique biochemical characteristics and the essential role of its structural domains [14,84]. The role and activity of PLCZ1 and PLCZ1 isoforms have been identified in mammalian and nonmammalian species, such as chicken and medaka fish [4,5,13,58,59,60,62,63,64,65,68,69,70,71,85]. Although minor sequence variations can significantly alter PLCZ1 function in a species-specific manner [4,86], PLCZ1 structure, size, and function appear to be conserved among mammals.

After gamete fusion, PLCZ1 is proposed to be released into the ooplasm to hydrolyze membrane-bound phosphatidylinositol-4,5-bisphosphate [PIns(4,5)P_2_] in intracellular vesicles into inositol-1,4,5-trisphosphate (InsP_3_) and diacylglycerol (DAG). In the endoplasmic reticulum, InsP_3_ binds to the inositol 1,4,5-trisphosphate receptors (InsP_3_R), resulting in conformational changes to its intrinsic Ca^2+^ channel and Ca^2+^ release from the endoplasmic reticulum into the ooplasm. In parallel, DAG activates protein kinase C (PKC), which results in the upregulation of several downstream events necessary for fertilization in a time-dependent manner [16,73,74,84,87]. The release of Ca^2+^ activates calcium/calmodulin-dependent protein kinase II (CaMKII), which inhibits the cytostatic factor EMI2 (CSF) by phosphorylation. The downregulation of CSF releases the inhibition of the anaphase-promoting complex/cyclosome (APC/C) and degrades the levels of cyclin B1 from the maturation-promoting factor (MPF) complex, composed of cyclin-dependent kinase I (CDK1) and cyclin B1 (CNB1). The reduction in CNB1 downregulates MPF, releasing the oocyte from meiotic arrest. Intracellular zinc ion (Zn^2+^) release occurs concurrent with the intracellular Ca^2+^ rise and also downregulates CSF (EMI2). In addition, Ca^2+^ upregulates PKC, which phosphorylates myristoylated alanine-rich C kinase substrate (MARCKS), inducing actin breakdown, and allowing for cortical granule exocytosis. The inactivation of mitogen-activated protein kinase (MAPK) by Ca^2+^ leads to pronuclear formation (Figure 1B) [88,89]. The initial communication between the sperm and oocyte after fertilization is still unknown. PLCZ1 would be active only upon entry into the ooplasm, and it is uncertain how the oocyte activates PLCZ1 [9]. The existence of a putative binding partner or receptor for PLCZ1 within the fertilized oocyte is critical for the characterization of the physiological mechanism of PLCZ1 for oocyte activation [76,88]. The fidelity of the pattern and overall duration of Ca^2+^ oscillations induced by PLCZ1 at fertilization are decisive factors for pre- and post-implantation embryo development [90,91]. Overall, the distinctive oscillatory pattern of Ca^2+^ rises and the coordinated downstream events triggered by PLCZ1 are sufficient to initiate the oocyte activation cascade and impact embryo competence.

Coordinated oscillations of Zn^2+^ after Ca^2+^ release have been found in oocytes after gamete–membrane fusion [92,93,94]. Fluctuations in the concentration and localization of Zn^2+^ play pivotal roles in oocyte maturation, fertilization, and embryogenesis [92,93,94,95,96,97,98,99,100,101,102]. In mouse oocytes used as a biological model, Zn^2+^ is the most abundant transition metal in the oocyte. The total cellular content of Zn^2+^ increases as the oocyte progresses from prophase I arrest to arrest at the metaphase of meiosis II (MII). The Zn^2+^ influx is required for the oocyte to progress through anaphase by binding the Zn^2+^-dependent factor EMI2 [103]. After fertilization, the total Zn^2+^ content in the MII oocyte markedly reduces, indicating the transition between the meiotic and mitotic cell cycles. The Zn^2+^ efflux occurs via the exocytosis of Zn^2+^-enriched cortical vesicles in a biological process termed “zinc sparks” (Figure 1B) [92,93,94,96,97]. To test whether bovine oocytes also undergo Zn^2+^ fluxes during oocyte activation, bovine oocytes were injected with bovine *PLCZ1* cRNA, ionomycin, and bull sperm. Bovine *PLCZ1* cRNA or ionomycin parthenogenetically activated bovine oocytes, inducing coordinated Zn^2+^ sparks with intracellular Ca^2+^ oscillations [104]. Bovine *PLCZ1* cRNA resulted in a few Zn^2+^ sparks immediately after inducing multiple Ca^2+^ rises. Bull sperm also induced a Ca^2+^ rise and Zn^2+^ sparks when the oocyte activation was successful after sperm injection [104]. A congruent response has been observed in mouse embryos produced by IVF [92,102]. The response of the oocyte to artificial activation via PLCZ1 closely mimics Ca^2+^ oscillations required for oocyte activation, being a more physiologic approach than most chemical oocyte activators [8,105,106] and, by implication, Zn^2+^ sparks. The Zn^2+^ spark has been demonstrated in cattle, humans, nonhuman primates, and mice, and it seems to be a conserved event during the fertilization of mammalian species examined to date [92,93,94,96,97,104]. Intracellular Ca^2+^ oscillations and Zn^2+^ sparks are linked, as Ca^2+^ rises immediately preceding rises in extracellular Zn^2+^ due to the exocytosis of cortical granules [92], a downstream event of the Ca^2+^ signaling triggered by PLCZ1 during oocyte activation.

During the fertilization of mouse oocytes, PLCZ1 accumulates in the interphase pronucleus due to a nuclear localization signal in the XY-linker. The translocation of PLCZ1 to the pronucleus occurs at 6 h after cRNA microinjection. The PLCZ1 accumulation results in the cessation of Ca^2+^ oscillations, but the oscillations resume after pronuclear envelope breakdown when the zygote enters mitosis [66,107]. In contrast to the mouse, PLCZ1 does not translocate to the pronucleus and remains in the cytoplasm and secondary polar body in bovine, equine, human, and rat zygotes [62,69,71]. These observations suggest differences in the mode of action of PLCZ1 among species.

**Figure 2:**
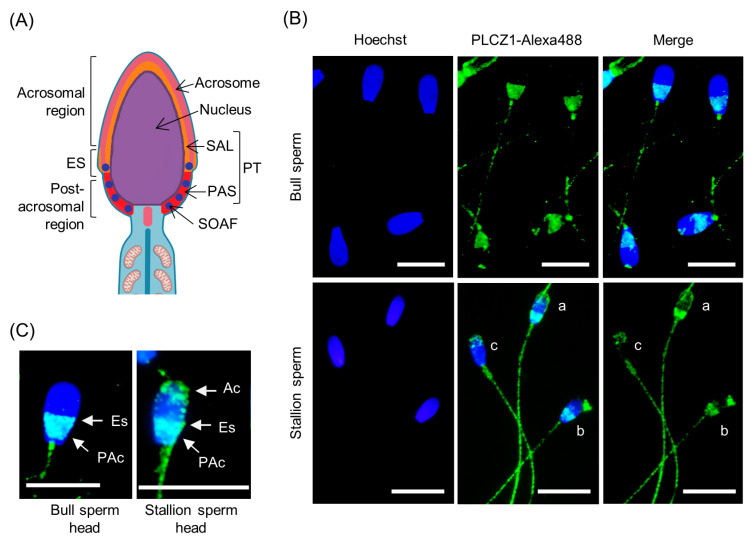
Representative diagram and immunofluorescence images of frozen-thawed bull and stallion sperm for PLCZ1 localization (**A**). The perinuclear theca (PT) surrounds the sperm nucleus and is divided into the subacrosomal layer (SAL) and the postacrosomal sheath (PAS). The putative sperm oocyte-activating factor (SOAF) is localized in the equatorial segment (ES) and postacrosomal sheath of the perinuclear theca [110,111]. Localization of PLCZ1 was assessed using a rabbit anti-PLCZ1 antibody of human origin (Santa Cruz Biotechnology, Dallas, TX, USA), goat anti-rabbit IgG-H+L-Alexa FluorTM488 (Invitrogen, Eugene, OR, USA), and Hoechst 33342 (Hoechst). (**B**,**C**) Bull sperm exhibit specific immunoreactivity for PLCZ1 in the equatorial segment (ES) and postacrosomal (PAc) region, but nonspecific labeling in the tail. (**B**,**C**) Stallion sperm exhibits specific PLCZ1 immunodetection in the acrosomal region (Ac), equatorial segment, and postacrosomal region of the sperm head, and, particularly, in the midpiece and principal piece of the tail. (**B**) Stallion sperm “a” shows all of the PLCZ1 localizations. However, sperm “b” and “c” lack PLCZ1 in the acrosomal and postacrosomal region, respectively. Image magnification: 1000×. Scale bars = 10 µm.

The localization of PLCZ1 on the sperm has been studied in various species to further define its function during fertilization. In mammalian species studied to date, PLCZ1 predominantly localizes in the acrosomal, equatorial, and postacrosomal regions of the sperm head [5,24,62,68,69,70,108] (Figure 2A), exhibiting dynamic patterns related to preservation state (fresh or frozen-thawed) or capacitation status (noncapacitated, capacitated, or acrosome-reacted) [61,63,64,67,109]. In human sperm, PLCZ1 was confirmed in a subcellular localization along the inner acrosomal membrane and in the postacrosomal area of the perinuclear theca by immunogold labeling and electron microscopy. The postacrosomal area of the perinuclear theca corresponds to the postacrosomal PLCZ1 localization in immunofluorescence images [108]. By definition, the sperm oocyte-activating factor should be present in the acrosome-reacted sperm head, likely in the equatorial segment or postacrosomal region [110]. The first component of the sperm cytosol to enter the ooplasm is the postacrosomal sheath of the perinuclear theca [110,111]. The perinuclear theca is a nonionic detergent-resistant and condensed cytosolic protein layer that forms the major cytoskeletal component in the sperm head. The perinuclear theca surrounds the sperm nucleus and is separated into the subacrosomal layer and postacrosomal sheath (Figure 2A), which are structurally and compositionally distinct [111]. The disassembly of the perinuclear theca containing the sperm-borne oocyte activating factor triggers oocyte activation [110,111]. The subcellular localization of PLCZ1 aligns with the requirement that the sperm-borne oocyte-activating factor should be localized in the postacrosomal area of the perinuclear theca to be strategically released after gamete fusion.

During the passage through the female reproductive tract, sperm undergo capacitation to acquire fertilizing capability for in vivo fertilization. Sperm undergo several biochemical events that lead to plasma membrane destabilization and hyperactivation. Capacitated sperm are capable of undergoing the acrosome reaction to penetrate the zona pellucida and reach the oocyte membrane. In noncapacitating conditions, sperm from hamsters, men, and mice exhibit PLCZ1 in the acrosomal, equatorial, and postacrosomal regions [67,68,109,112]. In noncapacitated bull sperm, PLCZ1 is located in the equatorial region of fresh sperm (Figure 2B,C) [64]. In noncapacitated stallion sperm, PLCZ1 is localized in the acrosomal and equatorial regions, and particularly in the connecting piece and principal piece of the tail (Figure 2B,C) [68,69]. Under capacitating conditions, PLCZ1 undergoes dynamic localization changes in sperm from hamsters, men, mice, and stallions. PLCZ1 in the sperm of these species is mostly detected in the equatorial segment and/or postacrosomal region, but acrosomal localization is almost absent [67,68,109,112]. In bull sperm, PLCZ1 migrates to the postacrosomal region and remains in this location and at the base of sperm neck after capacitation and acrosome reaction [64]. The dynamic changes of PLCZ1 localization during capacitation can be explained by the interaction of PLCZ1 with other sperm components. Dynamic localization changes of PLCZ1 across species may reflect the functional importance of acrosomal PLCZ1 for capacitation and/or the acrosome reaction or events other than its role as an oocyte-activating factor after gamete fusion.

In functional localization studies of porcine sperm, PLCZ1 can be retrieved by sonication and/or freezing–thawing cycles; in addition, several other proteins are also extracted in the cytosolic sperm extract [59,70,79,113,114]. Injections of porcine cytosolic sperm extract into mouse oocytes result in PLC activity, triggering Ca^2+^ oscillations and supporting oocyte activation. After extraction, sperm lose their membranes but retain their capacity to trigger Ca^2+^ release and oocyte activation [59,70,115]. Alkaline carbonate extraction, which is an extraction method using high pH, is frequently used to disrupt lipid membrane vesicles and collect membrane-associated proteins. Porcine sperm exposed to alkaline carbonate extraction lose their capability to initiate Ca^2+^ oscillations. However, the high-pH sperm extract retains Ca^2+^ oscillatory activity with less full-length PLCZ1 and with the presence of enzymatically active products, which are likely the result of partial PLCZ1 proteolysis [59,79]. In porcine sperm, extraction by repeated freeze–thaw cycles results in a sperm extract containing abundant acrin1, an acrosomal marker, and scarce MN13, a perinuclear theca marker. Nonionic detergent extraction by Triton X-100 leads to undetectable acron1 and the marked presence of MN13. This differential sperm component extraction indicates that the soluble fraction obtained by freezing contains primarily acrosomal components, and the nonionic detergent extraction removes perinuclear theca components. However, both soluble and nonionic detergent extracts contain enzymatically active PLCZ1. To confirm that PLCZ1 is extracted from the perinuclear theca, sperm exposed to cytosolic extraction retain substantial PLC activity in the sperm head, which is only suppressed by high pH to remove perinuclear theca components [70]. This distinctive extraction of sperm proteins suggests that PLCZ1 is distributed in different sperm compartments. A proportion of PLCZ1 is rather insoluble and restricted to the perinuclear theca, and from this oocyte-penetrating assembly localization, it can be released to trigger oocyte activation [59,70,116].

The solubility and relative quantity of PLCZ1 in an individual sperm varies among species, possibly influencing the ability to induce Ca^2+^ release and oocyte activation. Equine and porcine sperm contain more PLCZ1 when compared to mouse sperm [59,68]. Similarly, equine PLCZ1 has higher Ca^2+^-releasing activity than mouse PLCZ1 [68,69]. PLCZ1 solubility, which might be mediated by the existence of a putative binding receptor for PLCZ1 within the fertilized oocyte [76,88], can also affect the initiation of Ca^2+^ oscillations. After gamete fusion, hamster and mouse sperm initiate Ca^2+^ oscillations within about 10 s and 1–3 min, respectively [117,118]. In the heterologous fertilization of zona-free hamster oocytes with mouse sperm, Ca^2+^ oscillations also start several minutes after gamete–membrane fusion [118]. In contrast, equine *PLCZ1* cRNA injected into mouse oocytes shows higher activity, triggering earlier and higher frequency Ca^2+^ oscillations than human and mouse cRNA [69]. At the same concentration, bovine *PLCZ1* cRNA is not capable of inducing Ca^2+^ release, showing lower specificity and activity in mouse oocytes [69]. Interestingly, human and mouse *PLCZ1* cRNA trigger an initial wider Ca^2+^ oscillation with overlaid spikes before returning to the baseline [69], which is the characteristic Ca^2+^ oscillation pattern observed in these species [4,5]. These findings imply that the rapid response of the oocyte is related to the sperm and may be explained by species-specific differences in PLCZ1 solubility and interactions within the oocyte [8].

## 4. Equine PLCZ1

Phospholipase C zeta 1 in stallion sperm is a 638 amino acid protein with a molecular weight of ~73 kDa. The primary structure of equine PLCZ1 has significant homology with porcine (82.5%) and human (82.1%) PLCZ1 and partial homology with bovine (79.4%) and murine (71.9%) PLCZ1. The XY-linker region exhibits the least regional homology among these species [68].

In ontogeny characterization in stallions, PLCZ1 was first observed in mature spermatogenic cells close to the lumen of the seminiferous tubules, corresponding to round spermatids. In round spermatids, PLCZ1 localizes over the developing acrosome and is enzymatically active in triggering Ca^2+^ oscillations if injected into mouse oocytes [68]. In elongating spermatids and developing sperm, PLCZ1 is localized in heads and tails [68]. In epididymal and noncapacitated fresh equine sperm, PLCZ1 localizes in the acrosomal, equatorial, and postacrosomal regions, connecting piece, and principal piece of the tail (Figure 2B,C) [68,69]. In capacitated and acrosome-reacted stallion sperm, the acrosomal localization, protein content, and proportion of sperm exhibiting positive labeling for PLCZ1 are reduced [68,112,119]. The localization of PLCZ1 in the principal piece of the tail has been consistently observed in boar and stallion sperm (Figure 2B) [55,58,68,69,70,120,121]. Notably, the microinjection of a single isolated stallion sperm tail induces Ca^2+^ oscillations in mouse oocytes [68,122], confirming that PLCZ1 located in the sperm tail is enzymatically active and suggesting a functional variation in equine sperm.

Frozen stallion sperm are frequently used in equine ICSI. After selecting individual sperm for microinjection, the remaining frozen-thawed sperm can be refrozen. Frozen sperm can also be extended at a low concentration and refrozen for future procedures [123,124]. The cold shock of freeze–thaw procedures and membrane lipid phase changes can cause sperm damage and permanent conformational changes of sperm-borne membrane proteins [125,126], altering sperm physiology and reducing sperm fertilizing potential [127]. The localization of PLCZ1 in stallion sperm is conserved after one or two freeze–thaw procedures [55,68,121]. However, the relative quantity and the proportion of sperm positively labeled for PLCZ1 are reduced in frozen-thawed sperm samples when compared to fresh samples, but no further reduction is observed after a second freeze–thaw cycle [121,128,129]. Cryopreservation causes a loss in sperm plasma membrane integrity, facilitating the release of soluble sperm-borne components such as PLCZ1 to the medium, and they can be detected in cryodiluents after sperm freezing and refreezing [121,128,130]. The PLCZ1 content of equine sperm is strongly associated with plasma and acrosomal membrane integrity. Using a flow cytometric assessment, stallion sperm positively labeled for PLCZ1 with intact and damaged plasma membranes had similar levels of PLCZ1, but sperm with damaged acrosomes exhibited less PLCZ1 than acrosome-intact sperm, independent of whether those sperm were fresh, frozen, or refrozen [121]. A similar response to cryopreservation occurs in human sperm. A reduction in protein quantity and proportion of sperm exhibiting positive immunolabeling for PLCZ1, in addition to a greater proportion of sperm with only postacrosomal localization [25,131,132]. These observations suggest that PLCZ1 is principally lost from the acrosomal localization when sperm membranes are damaged by cryopreservation. In protein extraction experiments, a substantial proportion of PLCZ1 is removed from fresh equine sperm by a nonionic detergent (Nonidet-40), but in the subsequent extraction using an ionic detergent (sodium dodecyl sulfate), PLCZ1 is not totally extracted. A nonextractable fraction is retained in the postacrosomal region of the sperm, demonstrating that PLCZ1 interacts with sperm surface structures and, to a lesser extent, with internal structures [121]. The multiple localizations in sperm compartments of PLCZ1 suggest that PLCZ1 plays a role in the acrosome reaction in addition to activating Ca^2+^ release [8,68]. Overall, cryopreservation is harmful to acrosomal and plasma membrane integrity and PLCZ1 retention, but a proportion of PLCZ1 is retained in a detergent-resistant structure of the postacrosomal region of equine sperm after cryopreservation, congruent to the perinuclear theca.

In attempt to reduce sperm cryodamage and loss of PLCZ1, frozen stallion sperm can be thawed up to 5 °C and then refrozen at a low temperature in cryodiluents containing glycerol and methylformamide, resulting in better sperm motility and survival than sperm thawed at 37 °C and refrozen at room temperature [124]. Although refreezing stallion sperm at a low temperature does not increase PLCZ1 retention when compared to frozen sperm [121], greater proportions of motile sperm allow for further sperm sorting. Sperm samples from fertile men sorted by density gradient centrifugation have a higher proportion of sperm positive labeled for PLCZ1 than before sorting [132]. Cryopreservation methods that enable increased sperm survival after thawing can facilitate further sperm sorting by swim-up, density gradient centrifugation, or microfluidics to obtain a sorted sperm subpopulation with an expected greater PLCZ1 for individual sperm selection for ICSI.

The specific activity and relative potency of sperm PLCZ1 differ among species [8,59]. The PLC enzymatic activity that results in Ca^2+^ release is higher in equine PLCZ1 than in PLCZ1 from bulls, humans, and mice. The microinjection of equine *PLCZ1* cRNA into mouse oocytes triggers earlier and more frequent Ca^2+^ oscillations when compared to oocytes injected with cRNA of human and mouse origin [68,69]. Despite being delayed by approximately 20 min, the optimal and efficient activation of mouse oocytes by equine *PLCZ1* mRNA is accomplished with tenfold lower doses than mouse *PLCZ1* mRNA [133]. In equine oocytes, mouse *PLCZ1* cRNA triggers Ca^2+^ oscillations that begin within 40 min after injection and are relatively short in duration and more variable in frequency among oocytes, resulting in parthenogenic cleavage. The Ca^2+^ oscillation pattern induced by mouse *PLCZ1* cRNA varies in a dose-dependent manner, but Ca^2+^ spike patterns are similar to those oscillations induced by the injection of a single equine sperm [91]. Similarly, the potency to induce Ca^2+^ oscillations of hamster and human cytosolic sperm extract was exceptionally different in mouse or human oocytes [134,135]. In mouse oocytes, Ca^2+^ oscillations are triggered with a hundred times less human *PLCZ1* cRNA than cRNA of mouse origin. Congruently, mouse *PLCZ1* cRNA is less effective at generating Ca^2+^ oscillations in human oocytes [4,136]. Differences in relative PLC enzymatic activity may be caused by differences in the sequence of the XY-linker region. This linker region in equine PLCZ1 shows greater homology with human (49.0%), bovine (48.1%), and porcine (47.4%) PLCZ1 and less homology with mouse (35.4%) PLCZ1 [68]. Potentially, equine PLCZ1 could serve as an artificial oocyte-activating agent to promote oocyte activation in ICSI programs in other species due to its high enzymatic activity, sequence homology, and ability to mimic the oocyte activation induced by a sperm.

The relative content of PLCZ1 in stallion sperm has been associated with fertility in vivo and in vitro. In a clinical program, stallions with low in vivo fertility also performed less efficiently after heterologous or homologous ICSI [56], suggesting the implication of a male infertility factor. In studies with a limited number of stallions, the relative PLCZ1 abundance in fresh sperm exhibited an inconsistency of results among stallions with a low (<30%) or high (>30%) pregnancy rate after in vivo fertility. Stallions with low pregnancy rates exhibited lower or similar PLCZ1 abundance when compared to stallions with high pregnancy rates. A stallion with a low pregnancy rate had adequate progressive motile and morphologically normal sperm for breeding but showed a reduced PLCZ1. Interestingly, another stallion with a low pregnancy rate exhibited defects in the localization of PLCZ1, with a partial or total lack of PLCZ1 in the acrosomal region and/or equatorial region, regardless of having intact acrosomes [122,137]. Correspondingly, in a larger cohort of frozen-thawed sperm samples from stallions with proven fertility in vivo, the relative content and proportion of positively labeled sperm for PLCZ1 exhibited a wide range of values, differing from stallion to stallion [55]. The fertilizing potential of stallion sperm can be tested by the injection of a single sperm into bovine or porcine oocytes to assess pronuclear formation and cleavage rates [56,138]. When oocytes were injected with frozen-thawed sperm from samples with low or high PLCZ1, oocytes injected with sperm from samples with low PLCZ1 consistently demonstrated lower cleavage rates after injection into bovine and equine oocytes [55,121,139]. Similarly, mare oocytes injected with frozen-thawed stallion sperm were 36.6% less likely to cleave when compared to oocytes injected with cooled sperm that, by implication, had higher PLCZ1 [121]. In men, sperm from fertile individuals used for ICSI or IVF displayed important variations in the relative quantity and predominant localization pattern for PLCZ1. In some cases, the PLCZ1 content and localization pattern of fertile men were similar to those of infertile men with recurrent oocyte activation failure [130,140]. In clinical equine ICSI, the male factor plays a critical role in the outcome, especially when sperm used for injections come from a heterogeneous population of stallions and from sperm samples frozen under varied conditions. The content and localization of equine PLCZ1 have intrinsic variations among stallions that can be exacerbated by sperm preservation procedures impairing cleavage success and embryo competence.

The regulation of *PLCZ1* expression in stallions and its association with fertility are largely unknown. There are a handful of reports on genes that affect stallion fertility. In Hanoverian stallions, single nucleotide polymorphisms (SNPs) within genes of prolactin receptor [141], inhibin beta A [142], follicle stimulating hormone [143], angiotensin converting enzyme [143], spermatogenesis associated 1 [144] and cysteine-rich secretory protein 3 [145] contributed to the estimated breeding values of the paternal component of the pregnancy rate per estrus cycle, showing a significant connection with stallion fertility traits [141,142,143,144,145]. In Thoroughbred stallions diagnosed with impaired acrosomal reaction and reduced fertility, the FK506 binding protein 6 (FKBP6) gene in chromosome 13 was identified as an impaired acrosomal reaction susceptibility locus. The gene belongs to the immunophilins family, which are implicated in meiosis, calcium homeostasis, clathrin-coated vesicles, and membrane fusion [146]. Despite the fact that it has not been studied, mutations of FKBP6 may indirectly affect equine PLCZ1 localization and function in stallion sperm. Recently, an SNP was located within intron 8 of the *PLCZ1* gene of chromosome 6 in Hanoverian stallions. The validation of polymorphisms found in a large population of stallions characterized three intronic SNPs within *PLCZ1*. These *PLCZ1* SNPs are associated with the paternal component of pregnancy rates as a male fertility trait. SNPs were proposed to be regulated by transcription factor-binding sites, or microRNAs. This finding confirms that *PLCZ1* is an important gene for stallion fertility [147]. However, *PLCZ1* mutations that affect stallion fertility under in vivo or in vitro conditions have not yet been documented.

Protein expression of PLCZ1 in stallion sperm is associated with ICSI success. Genetic screening and protein evaluation for PLCZ1 could be markers for stallion selection to predict fertility in vivo and in vitro. Equine PLCZ1, due to its high PLC activity, could have value for artificial oocyte activation treatment, closely mirroring physiological events triggered by sperm injection.

## 5. Bovine PLCZ1

Bovine PLCZ1 is a 70–72 kDa protein compound of 634 amino acids and has a high homology with porcine PLCZ1 (83.0%) and lesser homology with equine PLCZ1 (79.4%) [13,68,105,148]. PLCZ1 is located in the equatorial and postacrosomal regions of bull sperm (Figure 2B,C); however, nonspecific immunoreactivity has also been detected in the acrosomal region and tail [13,63,64]. In some studies, PLCZ1 has been detected in the tail of noncapacitated, capacitated, and acrosome-reacted fresh or frozen-thawed bull sperm [61,64], although no determination was made as to the enzymatic activity or nonspecific immunoreactivity of PLCZ1 localized in the tail. However, the expected subcellular localization of PLCZ1 in the perinuclear theca has not yet been confirmed in bull sperm.

The capacitation status of bull sperm is associated with PLCZ1 modifications. Bovine PLCZ1 migrates to the postacrosomal region and remains in this location and at the base of the neck after capacitation and the acrosome reaction [64]. The interaction of PLCZ1 with other sperm molecules and cellular structures can explain the dynamic changes in PLCZ1 localization caused by capacitation. A sperm-specific Na/K-ATPase, ATP1A4, was shown to interact with PLCZ1 for PLC-tyrosine phosphorylation during in vitro capacitation, upregulating PLCZ1 activity. In somatic cells, the ATP1A1 subunit forms a signaling complex with PLC-gamma-1 and IP3 receptors to form a scaffold [149]. In rat and bull sperm, ATP1A4 is involved in the regulation of motility and capacitation events [150,151,152,153]. In noncapacitated bull sperm, ATP1A4 is localized in the entire head, and PCLZ1 is located in the acrosomal region; but, after capacitation, both enzymes undergo dynamic changes and colocalize in the postacrosomal region [63,154]. The ATP1A4–PLCZ1 interaction upregulates PLC activity via a mechanism that involves ATP1A4, contributing to capacitation events such as tyrosine phosphorylation, hyperactivation, and F-actin formation [63]. In noncapacitating conditions, bovine PLCZ1 interacts with G-actin rather than F-actin. Under capacitating conditions, PLCZ1 and actin migrate together to the postacrosomal region in the bull sperm, but PLCZ1 progressively loses its affinity for F-actin. As capacitation and the acrosome reaction continue, G-actin decreases, PLCZ1 affinity for F-actin declines, and more PLCZ1 is released into the extracellular medium [64]. In bull sperm, PLCZ1 localization and interaction with other sperm components could indicate that PLCZ1 has a functional role in capacitation and/or the acrosome reaction or events other than oocyte activation.

Species-specific differences in PLCZ1 activity appear to be evident when research models are used to characterize bovine PLCZ1 activity during fertilization. Oocyte activation and embryo development after bovine ICSI are limited [104,106,155]. The causes of ICSI failure include the inability of the bull sperm to undergo nuclear decondensation and pronuclei formation [156], impaired function of the microtubule-organizing center [157], and the incapacity of the bull sperm to consistently initiate Ca^2+^ oscillations in bovine oocytes [106]. Under experimental conditions, bull sperm can initiate Ca^2+^ oscillations and pronuclear formation after injection into mouse oocytes. When bull sperm are subjected to high pH-protein extraction, bull sperm lose PLCZ1 and ability to induce Ca^2+^ oscillations [13]. However, the high-pH extracted fraction retains Ca^2+^ oscillatory activity when injected into mouse oocytes, demonstrating that the high-pH extraction solubilizes PLCZ1 without eliminating its enzymatic activity [13]. The enzymatic activity of bovine PLCZ1 seems to be lower than that of other species. Under the same conditions, equine, human, and mouse *PLCZ1* cRNA trigger Ca^2+^ oscillations in mouse oocytes, but bovine *PLCZ1* cRNA does so at a lower rate or none at all [62,69]. In contrast, the microinjection of bovine or mouse *PLCZ1* cRNA into mature bovine oocytes is able to induce sperm-like Ca^2+^ oscillations in a dose-dependent manner, resulting in embryo development at rates comparable to artificial activation. Noticeably, less bovine *PLCZ1* cRNA is required to achieve optimal oocyte activation and higher InsP3R degradation in bovine oocytes than mouse cRNA, suggesting that bovine PLCZ1 has higher enzymatic potency in homologous oocytes than mouse PLCZ1 [86]. Based on cross-species research [13,62,69,71,91], species-dependent variations facilitate a precise regulation of the quantity and attributes of PLCZ1 released by the sperm to match the sensitivity of the receiving oocyte to efficiently trigger Ca^2+^ oscillations to induce oocyte activation.

The production of bovine embryos by standard in vitro fertilization (IVF) methods is very successful among large livestock species. Approximately 80% of bovine oocytes fertilized using IVF cleave and begin embryo development, and up to 40% of fertilized oocytes reach the blastocyst stage [158]. In contrast, the success of bovine ICSI is still limited [104,106,155], although bovine *PLCZ1* cRNA can trigger Ca^2+^ oscillations and promote parthenogenetic embryo development under experimental conditions [86]. Bull sperm injected into bovine oocytes are unable to consistently evoke Ca^2+^ oscillations for oocyte activation [106] and undergo nuclear decondensation and pronuclei formation [156]. The failure of consistent oocyte activation may be explained by the high rigidity of the perinuclear theca observed in bovine sperm, which interferes with the solubilization of the perinuclear theca contents [110,159,160] and, by implication, the release of PLCZ1 into the ooplasm. Bull sperm has only protamine 1, and the nuclear chromatin is very stable with the maximum number of disulfide cross links [161], resulting in high nuclear stability when compared to human and mouse sperm [162]. Nuclear chromatin stability contributes to the difficulty of nuclear decondensation and remodeling after bovine ICSI [155]. In an attempt to address these difficulties, frozen-thawed bull sperm were treated before homologous ICSI with lysolecithin or Triton X-100 to facilitate acrosomal and plasma membrane destabilization. Acrosomal and plasma membrane integrity and PLCZ1 content were reduced in a dose-dependent manner, but destabilized bull sperm resulted in higher cleavage and blastocyst rates than untreated sperm after sperm injection and artificial activation with ionophore and cycloheximide [163]. Apparently, sperm chromatin decondensation is blocked due to the presence of the acrosome and related substructures, which delays the mechanisms required for chromatin remodeling before pronuclear formation [164]. Thus, the removal of acrosomal components could assist sperm nuclear remodeling for oocyte activation [165]. This condition appears to be species-dependent, as the same membrane destabilization protocol using lysolecithin or Triton X-100 did not affect PLCZ1 in rat sperm, and acrosomal removal is not required for human and mouse ICSI [165,166]. The loss of PLCZ1 may be explained by the use of membrane destabilization reagents that can remove PLCZ1 from the equatorial region localization in bull sperm as used for the extraction of cytosolic sperm content [113,114]. For a successful bovine ICSI, artificial activating treatments to induce Ca^2+^ oscillations and rescue oocyte activation are required to compensate for the deficit of PLCZ1 when membrane-destabilizing procedures are used to facilitate the nuclear decondensation and remodeling of bull sperm.

The regulation of PLCZ1 expression in bulls is not well known. No genetic mutations in bovine PLCZ1 have been reported to compromise fertility in vivo or in vitro. However, specific polymorphisms in the PLCZ1 gene have been identified. In a population of black and red Holstein bulls, the resulting genotypes of PLCZ1 polymorphisms did not show any effect on sperm quality traits [167]. In contrast, two novel variants of PLCZ1 were identified in Chinese Holstein bulls. These variants contribute to the transcriptional activity of the PLCZ1 promoter, resulting in phenotype diversity and changes in semen quality traits [168]. Also in Chinese Holstein bulls, a novel splice variant of PLCZ1 was identified in testis tissue. The protein sequencing analysis revealed amino acid deletions that resulted in altered transcription of the Y and C2 domains, impairing catalytic and lipid binding functions [148]. These findings confirm the existence of genotype and, consequently, phenotype diversity for PLCZ1 in bulls. However, studies need to be conducted to investigate the association between these genetic variants of *PLCZ1*, protein expression, and fertility in cattle.

Recently, sperm mRNA characterization has gained considerable interest as a potential diagnostic tool to select bulls for reproductive performance. The mRNA population existing in a sperm is thought to be a representation of past cellular events during spermatogenesis [169,170,171,172]. Proteins in bull sperm such as adenylate kinase 1 (AK1), integrin beta 5 (IB5), nerve growth factor (NGF), tissue inhibitors of metalloproteinases 2 (TIMP2), lactate dehydrogenase C 1 (LDHC), small nuclear ribonucleoprotein polypeptide N (SNRPN), and the outer dense fiber of sperm tails 2 (ODF2) are associated with sperm functionality and fertility in vivo. The expression of mRNA coding for these proteins and PLCZ1 is associated with sire conception rate and is differentially expressed among bulls with low, average, and high in vivo fertility. A regression model that included *AK1, IB5, TIMP, SNRPN2*, and *PLCZ1* mRNA accounted for 97.4% of the variance in sire conception rates, which reflects the potential to sire calves [61]. The genetic screening of bulls and their sperm is a powerful tool to diagnose subfertility and predict the fertility potential of bulls that are frequently selected by production traits.

Bovine PLCZ1 has close homology to porcine and equine PLCZ1 and shares many similarities with other mammalian PLCZ1. Genetic screening of bulls and bovine sperm demonstrates that PLCZ1 influence fertility in vivo. Genetic screening of bulls for PLCZ1 and assessment of protein expression could be tools for bull selection to predict fertility in vivo and in vitro. Because bovine ICSI is not efficient, the role of PLCZ1 for fertility in vitro is less clear. However, under optimized ICSI conditions, PLCZ1 could be a valuable agent for artificial oocyte activation that closely mimics physiological events during fertilization.

## 6. The Evaluation of PLCZ1 as Diagnostic Tool to Predict Fertilization

The association between sperm PLCZ1 and male infertility in assisted fertilization has been experimentally and clinically established in humans and horses [13,19,24,25,26,27,28,29,30,31,32,33,34,35,36,37,38,39,40,41,42,43,44,45,46,55,122]. In human sperm, the absence, reduced quantity, or altered localization patterns of PLCZ1 are frequently associated with oocyte activation failure after ICSI [25,29,40]. The proportion of a sperm population displaying positive labeling for PLCZ1 associates with cleavage rates in humans and horses [43,45,55,121]. Therefore, the characterization and assessment of PLCZ1 in sperm samples could be a useful tool as a biomarker to diagnose and predict sperm fertilizing potential [32,33,55,173,174].

Genetic analysis of the male and immunodetection for PLCZ1 in sperm samples have been developed as diagnostic tests to evaluate mutations and abnormal protein expression (Table 1). Genomic DNA and bioinformatics analysis have been used for the identification of abnormalities in the *PLCZ1* sequence. To date, 27 exonic variations in *PLCZ1* have been identified in humans and associated with reduced fertility or oocyte activation failure after ICSI [30]. Most *PLCZ1* variants impair at least one of the domains involved in catalytic and regulatory PLC activity, producing a partial or complete failure to induce Ca^2+^ oscillations [30]. In human studies, 30–40% of oocyte activation failure cases involve a *PLCZ1* mutation [31,175]. Not all *PLCZ1* mutations identified to date result in apparent protein dysfunction or altered localization patterns [30,31,140]. Quantitative real-time PCR has been used to detect *PLCZ1* mRNA expression in sperm. The lower *PLCZ1* mRNA expression from morphologically normal and globozoospermic sperm samples from men is associated with low fertilization rates after ICSI [25,108,174,176]. Sperm mRNAs are thought to be a representation of cellular events during spermatogenesis [169,170,171,172] and to be important for fertilization and phenotypic traits of the progeny [177,178]. However, this assay does not identify protein content or localization patterns in the sperm. Overall, oocyte activation failure can occur as a consequence of a wide variety of *PLCZ1* mutations [31,130,140] that are diagnosed by genetic analysis, but genetic screening does not reveal translational changes that impair protein function, structure, or distribution for successful oocyte activation (Table 1).

Immunodetection is established on the premise that a primary antibody precisely recognizes an epitope of the target protein to produce a specific signal, and a labeled-secondary antibody binds to the primary antibody. The label of the primary–secondary antibody complex can be detected by different techniques such as immunofluorescence, immunocytochemistry, immunohistochemistry, immunoblotting, or enzyme-linked immunoassay (ELISA). In numerous studies of PLCZ1, immunofluorescence microscopy has been used to characterize PLCZ1 in sperm of different species, allowing for the imaging of protein localization, distribution patterns, and the relative quantification of protein content in a limited number of sperm. In addition, some sperm structures can be parallelly evaluated for colocalization studies. Advantageously, fluorescence emitted by the PLCZ1 primary–secondary antibody complex and other sperm attributes can be simultaneously measured in thousands of sperm cells by flow cytometry to evaluate sperm heterogenicity [55,119,121,179,180]. In a recent study, a commercial ELISA was used to quantify PLCZ1 in equine sperm from fertile stallions, showing congruent results with immunofluorescence and immunoblotting, although the ELISA used a different but specific antiPLCZ1 antibody [121]. ELISA is more sensitive and specific than indirect immunofluorescence and has comparable diagnostic accuracy. ELISA is faster, simpler, and less dependent on the operator [181]. Immunodetection techniques are subjected to intra- and inter-laboratory variation that interferes with objective comparisons and the homologation of results (Table 1). The development of techniques and protocols to reduce assessment variation is needed to conduct PLCZ1 analysis as a comparable and standard procedure for clinical use.

The total relative sperm content and localization patterns for PLCZ1 are routinely assessed by immunofluorescence and immunoblotting. In men with high fertility rates after ICSI, the most frequent PLCZ1 localization pattern is the equatorial and postacrosomal localization [25,29,40,43,140,173]. In addition, a dispersed localization covering the sperm head has also been described as a predominant PLCZ1 localization in human studies [43,45,130]. In contrast, a lack of PLCZ1 in the equatorial region of human sperm is linked to total oocyte activation failure [25,29,40,43,130,173]. As expected, the total relative content or proportion of sperm positive-labeled for PLCZ1 was higher in fertile men when compared to men with low or failed fertilization after ICSI or IVF [32,43,45,173]. However, in some studies, the proportion of sperm exhibiting positive labeling or fluorescence intensity for PLCZ1 did not differ between subfertile and fertile men subjected to ICSI or IVF [45,180]. The disparity of results can be explained by the PLCZ1 assessments in human sperm, as current assays exhibit a wide range of total levels or predominant localization patterns. The considerable variance in sperm PLCZ1 resultants of assessments of infertile men subjected to ICSI cannot be efficiently discriminated from fertile men, leading to a weak or null association with fertility after ICSI [130,140,180]. Another variable in assessing PLCZ1 is the heterogeneity of the total sperm population within a sperm sample. PLCZ1 localization patterns vary depending on the acrosomal and plasma membrane integrity of the sperm, indicating a cofounding association with the initial sperm quality in a sample. In human and equine studies, sperm subpopulations with intact acrosomal and plasma membranes have a different proportion of localization patterns, relative quantity, and proportion of sperm positive for PLCZ1 than subpopulations with disrupted acrosomal and plasma membranes [43,55,109,121,140]. However, oocyte activation failure can occur even when the relative quantity and localization patterns of PLCZ1 seem to be within a normal range, suggesting that quantification and localization assessments for PLCZ1 are necessary but not absolute methods to determine sperm oocyte-activating competence [140,180]. The large variation in the indirect quantification of PLCZ1 and the lack of standard methodologies for immunofluorescence techniques limit the diagnostic utility and predictive value of immunodetection for PLCZ1. A standard procedure that accounts for species-specific differences is required to establish objective and comparable PLCZ1 assessments across laboratories.

The immunodetection of sperm PLCZ1 relies on antibody specificity and sensitivity. Despite PLCZ1 being conserved among mammalian species [4,5,13,58,59,60,62,63,64,65,68,69,70,71], minor species-specific variations seem to be enough to change the protein recognition of antibodies. The nature of antibodies may explain the variation in the detection of PLCZ1 localization patterns and nonspecific reactivity observed among existing studies. For instance, antibodies raised against PLCZ1 of human, murine, or porcine origins specifically recognize sperm PLCZ1 from the original species and others such as bovine or equine, but with nonspecific labeling in the sperm tail in the case of the bull [13,182]. Antibody specificity and cross-immunoreactivity can be proven following immunoblotting, immunofluorescence, and peptide-blocking experiments. However, immunofluorescent visualization efficacy can be poor, possibly due to the steric or conformational occlusion of native PLCZ1, impeding antibody access [130]. In an attempt to improve the visualization of PLCZ1 in human, mouse, and boar sperm, antigen unmasking-retrieval techniques can be conducted, enhancing the epitope availability [183,184]. Fixation methods using methanol instead of aldehyde following the antigen unmasking protocol can also enhance the visualization effectiveness of human and mouse PLCZ1 [184]. However, depending on laboratory practices, antigen unmasking protocols can be ineffective in enhancing immunofluorescence visualization of PLCZ1 in human sperm [185]. These findings can reflect differences in the affinity and specificity of antibodies and intra- and inter-laboratory methodologies, exposing the lack of standardization for PLCZ1 analysis. However, efforts need to be oriented toward the production of high-affinity and species-specific monoclonal antibodies against PLCZ1, and the establishment of a standard procedure is needed before quantification methodologies can be a reliable diagnostic and prognostic tool for clinical applications.

## 7. The Potential Therapeutic Role of PLCZ1 for Artificial Oocyte Activation

In cases of repeated oocyte activation failure in humans, oocyte Ca^2+^ analysis and heterologous and homologous ICSI can be implemented to indirectly test the Ca^2+^ oscillatory activity and activation rate of problematic sperm samples [186,187,188]. In humans, the preferred heterologous ICSI test is the mouse model to assess the activation rate by cleavage after sperm injection into mouse oocytes [186]. In equines, stallion sperm can be injected into bovine or porcine oocytes to assess oocyte activation [54,55,56,138]. In cases of fertility failure after ICSI, fertilization rates increase with artificial oocyte activation after sperm injection [188,189], implying that chemical artificial activation or exogenous PLCZ1 can be helpful in some cases related to male PLCZ1 alterations (Table 2).

The clinical choice for artificial oocyte activation in humans is Ca^2+^ ionophores, which artificially restore a sufficient Ca^2+^ release at the time of ICSI to trigger oocyte activation events and embryo development [190,191]. When associated with infertility in men, oocyte activation failure after ICSI has been attributed to *PLCZ1* mutations or protein alterations [13,19,24,25,26,27,28,29,30,31,32,33,34,35,36,37,38,39,40,41,42,43,44,45,46] in 30 to 40% of failures [31,40,178]. Interestingly, the injection of sperm from men with *PLCZ1* mutations along with ionomycin as an artificial activation agent resulted in fertility rates of >60%, which are comparable to fertile men and result in pregnancies and live births [31,37,192]. Altered Ca^2+^ oscillations after fertilization in mouse oocytes can exert potentially deleterious effects on gene expression and embryo development [193]. However, the gene expression patterns of human oocytes artificially activated after ICSI are closer to IVF than to ICSI alone, suggesting that artificial oocyte activation effectively mimics the genetic events initiated by sperm entrance [194], resulting in pregnancy and the normal neurodevelopmental outcome of offspring [191,195]. Importantly, fertilization can be artificially rescued in oocytes that do not activate as a result of the dysfunction of sperm PLCZ1.

**Table 2:** Use of artificial oocyte activation (AOA) for fertilization failure after intracytoplasmic sperm injection (ICSI) when related to male factors associated with *PLCZ1* gene mutations or sperm PLCZ1 protein alterations. IB: immunoblotting. IF: immunofluorescence.

Species	Features Analyzed	Outcome
Human [33]	IF for PLCZ1 localization patterns Genomic analysis for *PLCZ1* mutations Fertilization after ICSI	Clinical trial. <30% PLCZ1-positive sperm associated with fertilization failure. Gene mutations impaired sperm theca formation and PLCZ1. AOA improved fertilization in PLCZ1-negative sperm. Limited to couples with a history of ≤10% fertilization.
Human [19]	IF for PLCZ1 localization patterns Electron microscopy Sperm chromatin structure analysis FISH for aneuploidy assessment Fertilization after ICSI	Clinical trial. Multiparametric analysis. Sperm PLCZ1 is associated with fertilization. Gene mutations impaired sperm theca formation and PLCZ1. AOA improved fertilization in PLCZ1-reduced or -negative sperm. Limited to globozoospermic patients.
Human [32]	IF for PLCZ1 localization patterns Fertilization after ICSI	Clinical trial. Higher sperm PLCZ1 was associated with high fertilization rates. AOA improved fertilization in PLCZ1-reduced sperm.
Human [37]	IB for PLCZ1 abundance qPCR for mRNA *PLCZ1* Genomic analysis for *PLCZ1* mutations Fertilization after ICSI	Clinical trial. *PLCZ1* mutations and impaired PLCZ1 protein was associated with male infertility. AOA improved fertilization in men carrying *PLCZ1* mutations.
Human [31]	IB for PLCZ1 abundance IF for PLCZ1 localization patterns Genomic analysis for *PLCZ1* mutations Fertilization after ICSI	Clinical trial. PLCZ1 absence related to fertilization failure. Gene mutations impaired PLCZ1 function. AOA improved fertilization in *PLCZ1* mutations and resulted in birth.
Human [25]	IB for PLCZ1 abundance IF for PLCZ1 localization patterns Genomic analysis for *PLCZ1* mutations Fertilization after ICSI	Experimental model. PLCZ1 absence related to fertilization failure. Gene mutations impaired PLCZ1 abundance, size, and function. AOA rescued oocyte activation using human PLCZ1-impaired sperm in a heterologous ICSI model.
Mouse [20]	IF for sperm exhibiting PLCZ1 Genomic analysis for *PLCZ1* knockout Fertilization after ICSI and births	Experimental model. *PLCZ1*-knockout sperm does not activate oocytes. Activation by mRNA *PLCZ1* or AOA rescued oocyte activation of *PLCZ1*-knockout sperm, resulting in births.
Mouse [50]	IF for sperm exhibiting PLCZ1 Gene knockout Fertilization after ICSI and births	Experimental model. *PLCZ1*-knockout sperm did not activate oocytes. Activation by mRNA *PLCZ1* rescued oocyte activation of *PLCZ1*-knockout sperm resulting in births.
Mouse [133]	Oocyte activation test Fertilization after ICSI using chemically inactivated sperm and births	Experimental model. PLCZ1-inactivated sperm did not activate oocytes. Activation by equine mRNA *PLCZ1* rescued oocyte activation of PLCZ1-inactivated sperm resulting in births.
Mouse [196]	IF for sperm exhibiting PLCZ1 Fertilization after ICSI using round spermatids	Experimental model. Activation by human mRNA *PLCZ1* plus auxin-inducible degron technology improved oocyte activation of round spermatids resulting in births.

In several animal models, the injection of *PLCZ1* cRNA provokes Ca^2+^ oscillations and induces oocyte activation in a dose-dependent manner [13,62,68,69,86,91,133,196,197]. Such a Ca^2+^ response is congruent with the events observed in embryos produced by IVF [92,102]. Prominently, mouse *PLCZ1* cRNA is capable of rescuing the oocyte activation of mouse oocytes also injected with human sperm devoid of PLCZ1 [182]. In an optimized procedure, mouse oocyte activation induced by the injection of human *PLCZ1* mRNA followed by a murine spermatid produced blastocyst development and births of live offspring at a higher rate than mouse *PLCZ1* mRNA [197]. To test the oocyte activating potential of equine PLCZ1 as a treatment, mouse sperm inactivated by high-pH treatment were injected into mouse oocytes with equine or mouse *PLCZ1* mRNA or strontium as a chemical oocyte activator. The blastocyst development was similar among activating treatments, but birth rates were slightly reduced when oocytes were activated by equine *PLCZ1* mRNA in comparison with mouse mRNA or strontium activation. These findings suggest that mechanisms related to the artificial induction of oocyte activation could influence developmental competence [133]. Notably, PLCZ1 from a different species can support embryo development after oocyte activation in a homologous ICSI model, serving as an artificial oocyte activation treatment.

The injection of the recombinant PLCZ1 protein should trigger Ca^2+^ oscillations. In fact, recombinant human PLCZ1 injected into human and mouse oocytes under experimental settings is capable of inducing Ca^2+^ oscillations and cleavage, reassembling the events initiated by a sperm after ICSI [11,198,199]. Moreover, the injection of recombinant human PLCZ1 supported embryo development up to the blastocyst stage in mouse oocytes [11]. The injections of heated-inactivated sperm with recombinant human PLCZ1, Ca^2+^ ionophore, or strontium into mouse oocytes were able to trigger Ca^2+^ oscillations, initiate oocyte activation, and promote embryo development at similar rates when compared to the control ICSI [200]. These findings suggest that PLCZ1-mediated oocyte activation by RNA or recombinant proteins can be an efficient therapeutic option. When compared to chemical activators, artificial PLCZ1-mediated oocyte activation closely replicates Ca^2+^ oscillations caused by sperm, making it a more physiologic method to treat oocyte activation failure [8,11,105,106,198,199,200]. However, further research is needed to determine whether such artificial activating treatments represent the safest therapeutic means for overcoming oocyte activation failure.

## 8. Conclusions

Clinical and experimental evidence demonstrate that PLCZ1 is the predominant sperm oocyte-activation factor in mammals. Genetic and protein alterations in PLCZ1 are directly associated with oocyte activation failure and low fertility. In humans, 30–40% of fertility failure after ICSI is caused by male infertility linked to impaired gene sequence or protein content, localization, and function of PLCZ1. Therefore, PLCZ1 assessments using accurate and repeatable tests could have a diagnostic and predictive value for sperm fertilizing potential and oocyte activation.

The role of PLCZ1 in assisted fertilization is critical. Men carrying PLCZ1 mutations associated with oocyte activation failures are able to have high fertility rates after ICSI and artificial oocyte activating treatment comparable to fertile men, achieving full development and births. The injections of *PLCZ1* mRNA from several mammalian species and recombinant PLCZ1 protein into oocytes induce Ca^2+^ oscillations and promote oocyte activation. Remarkably, PLCZ1 has been shown to be a successful artificial oocyte-activating agent in animal models, not only rescuing fertilization but also supporting embryo and full development until birth. The intervention with PLCZ1 has the potential to be a therapeutic application with a more physiologic approach to treat oocyte activation failures than chemical activators.

In cattle and horses, current breeding techniques can minimize the role of PLCZ1 for in vivo fertility. However, genetic variants and the altered protein expression of PLCZ1 may influence sperm quality traits in bulls and stallions that impact fertility in vivo and in vitro. Among stallions, the PLCZ1 content of sperm samples varies, and sperm samples with low PLCZ1 are associated with low cleavage rates after ICSI. Equine PLCZ1 has higher enzymatic activity, which has been advantageously used as a therapeutic agent to assist Ca^2+^ release after sperm injection in animal models. Conversely, the success of bovine ICSI is poor due to the inconsistent potential of the bull sperm to trigger Ca^2+^ oscillations and undergo nuclear decondensation after ICSI. The assessments of PLCZ1 and its use as an oocyte-activating treatment can be tools to improve assisted fertilization outcomes in cattle and horses. Further research is needed to determine whether PLCZ1 as an artificial oocyte-activating treatment is a physiological, efficient, and safe method for improving assisted fertilization in cattle and horses.

## Figures and Tables

**Table 1:** Assays for direct and indirect assessment of PLCZ1 from sperm used for assisted fertilization.

Assay	Features Analyzed	Advantages	Disadvantages
Genetic analysis	Genomic DNA detects *PLCZ1* mutations. Mutations associate with fertilization failure.	Characterization of *PCLZ1* mutation in individuals or families for eligibility for oocyte activation treatment. Used in clinical trials	No information on protein content, distribution, or function.
Quantitative polymerase chain reaction	*PLCZ1* mRNA expression associates with fertilization rates.	Characterization of *PCLZ1* expression in sperm. Used in clinical trials.	No direct information on PLCZ1 protein. Relatively high cost.
Immunofluorescence	Protein abundance and distribution associate with fertilization rates.	Relatively low cost and rapid to perform. Used in clinical trials.	Evaluation of hundreds of sperm. Relies on antibody specificity. Differences inter-laboratory.
Immunoblotting	Relative protein abundance associates with fertilization rates.	Relatively low cost and rapid to perform. Used in clinical trials.	Proportional evaluation of sperm proteins. Relies on antibody specificity. Differences inter-laboratory.
Flow cytometry	Relative protein abundance associates with fertilization rates.	Relatively low cost and fast to perform. Evaluation of thousands of sperm. Used in clinical trials.	Relies on antibody specificity. Differences intra- and inter-laboratory. Requires flow cytometer.
Enzyme-linked immunoassay (ELISA)	Assessment of protein quantity	Relatively low cost. Fast to conduct in batches to reduce variation.	Relies on antibody specificity. Has not been tested in clinical trials.

## Data Availability

Data are contained within the article.
